# Epidemiology of invasive pneumococcal disease in Southwest Sweden during the first eleven years after the introduction of general childhood pneumococcal vaccination

**DOI:** 10.1371/journal.pone.0352333

**Published:** 2026-06-29

**Authors:** Tor Härnqvist, Karin Bergman, Erik Backhaus, Mats Dahl, Helena Kolberg, Caroline Ström Turesson, Malin Olander, Staffan Nilsson, Rune Andersson, Susann Skovbjerg, Johanna Karlsson

**Affiliations:** 1 Department of Infectious Diseases, Institute of Biomedicine, Sahlgrenska Academy, University of Gothenburg, Gothenburg, Sweden; 2 Department of Infectious Diseases, NU Hospital Group, Trollhättan, Region Västra Götaland, Sweden; 3 Department of Infectious Diseases, Södra Älvsborg Hospital, Borås, Region Västra Götaland, Sweden; 4 Department of Infectious Diseases, Skaraborg Hospital, Skövde, Region Västra Götaland, Sweden; 5 Närhälsan Management Group, Gothenburg, Region Västra Götaland, Sweden; 6 Department of Clinical Microbiology, Sahlgrenska University Hospital, Gothenburg, Region Västra Götaland, Sweden; 7 Department of Primary Health Care, Kungsbacka, Region Halland, Sweden; 8 Department of Laboratory Medicine, Institute of Biomedicine, Sahlgrenska Academy, University of Gothenburg, Gothenburg, Sweden; University of Glasgow School of Health and Wellbeing, UNITED KINGDOM OF GREAT BRITAIN AND NORTHERN IRELAND

## Abstract

**Background:**

Invasive pneumococcal disease (IPD) still causes significant morbidity and mortality. In this study, we describe incidence, risk factors, manifestations, and outcome of IPD in Southwest Sweden during the first eleven years after the introduction of conjugate pneumococcal vaccines in the childhood vaccination program in 2009.

**Methods:**

Clinical data from 2,288 consecutive episodes of IPD in Region Västra Götaland, Sweden during 2009–2019 were retrospectively collected from medical records. Incidence rates were calculated using population data from the same period. The results were compared to data from three previous studies from the same geographical area with a total follow-up of 56 years.

**Results:**

The incidence of all IPD episodes in 2009–2019 was 12.8/100,000/year. A very high IPD incidence was seen in patients with multiple myeloma (1,497/100,000) and chronic lymphocytic leukemia (505/100,000). Meningitis occurred in 26% of the IPD episodes in children <2 years compared to 4.3% in the age group ≥65 years (12/46 versus 60/1,403; *p* < 0.001). The opposite was found for pneumonia, which accounted for 22% of the IPD episodes in children <2 years compared to 77% among the elderly (10/46 vs. 1,085/1,403; *p* < 0.001). In 604 IPD episodes (26.4%), one or more complications were observed, most commonly parapneumonic effusion and empyema. When data were compared with the previous study period, the IPD incidence in children <2 years declined from 22.5 in 1996–2008 to 10.7 per 100,000 in 2009–2019, while only a modest reduction was observed in adults ≥65 years (from 45.0/100,000 to 41.2/100,000). The overall case fatality rate (CFR) increased from 9.9% in 1996–2008 to 12.9% in 2009–2019, which could be explained by increased patient age and underlying comorbidity.

**Conclusions:**

A substantial decrease in IPD incidence was seen in infants and young children but not in the elderly during the first eleven years after the introduction of the general childhood pneumococcal vaccination program. Patients with hematological malignancies remain a high-risk group of IPD.

## Introduction

*Streptococcus pneumoniae* (the pneumococcus) was estimated to cause more than 800,000 deaths in 2019 worldwide and was the bacterial pathogen associated with the most deaths among children younger than 5 years [1]. Invasive pneumococcal disease (IPD) with associated bacteremia and/or meningitis most commonly affects individuals of high or very low age. Chronic diseases, including pulmonary disease, cardiovascular disease, and various immunosuppressive conditions, add to the risk [2,3].

More than 100 pneumococcal serotypes with different potential to cause IPD have as yet been described [4,5]. In 2009, the 7-valent pneumococcal conjugate vaccine (PCV7) was introduced in the Swedish childhood vaccination program, later replaced by PCV13 and/or PCV10. Adherence to the childhood immunization program in Sweden is high, with a pneumococcal vaccine uptake above 96% in all infants from 2011 onwards. In 2019, the vaccine uptake in Swedish children aged 2 years was 97.1% [6]. PCV13 was approved for adults in Sweden in 2011. Since 2016, pneumococcal conjugate vaccines have been recommended for adults with specified predisposing factors, in addition to the pneumococcal polysaccharide vaccine (PPSV23). International studies have demonstrated a decline in IPD incidence following the introduction of routine infant vaccination [7,8]. In a Swedish context, a herd immunity effect was observed among older children and adults during the first eight years after PCV introduction. Among the elderly, however, the reduction in IPD caused by pneumococcal vaccine serotypes was largely offset by replacement with non-vaccine serotypes [9]. We have previously demonstrated this pattern for the first seven years after PCV introduction in Region Västra Götaland [10].

This study presents clinical data of IPD epidemiology in Southwest Sweden during the first eleven years after the introduction of conjugate pneumococcal vaccination in the general childhood vaccination program, including IPD incidence, risk factors, manifestations, disease severity, complications, and case fatality rate (CFR), with comparison to data obtained from the same region in the pre-PCV era.

## Materials and methods

### Study design and patient identification

This is a retrospective study of invasive pneumococcal disease among all residents in the Region Västra Götaland, Southwest Sweden, between 1 January 2009 and 31 December 2019. In a previous study, we characterized all IPD episodes occurring in the same geographic area between 2009 and 2015 in relation to pneumococcal serotype distribution [10]. The mean population of the area was 1,640,000 during the study period. PCV7 was introduced in the regional childhood vaccination program in January 2009, replaced by PCV13 in January 2010, and PCV10 in February 2015 (three doses at 3, 5, and 12 months of age). Catch-up vaccinations in older children were not performed.

The patients were identified from the laboratory information systems at the clinical microbiological laboratories at Sahlgrenska University Hospital, NU Hospital Group, Södra Älvsborg Hospital, and Skaraborg Hospital, all located in Region Västra Götaland. These laboratories served all hospitals in the region. Data on invasive pneumococcal isolates were collected from laboratories on June 8, 2015, May 23, 2016, and August 1, 2020. An IPD episode was defined by the detection of *S. pneumoniae* by culture and/or PCR in a specimen from a normally sterile body site, including blood, cerebrospinal fluid (CSF), synovial-, pleural-, peritoneal-, or pericardial fluid, or the vitreous body.

The study was approved by the Regional Ethics Committee of Gothenburg (no. 123–15, T351-16) and the Swedish Ethical Review Authority (no. 2020–03453). The ethics committee did not require individual consent due to the retrospective study design.

### Medical data and definitions of clinical manifestations

Clinical and demographic data, including pneumococcal vaccination status, were retrieved from medical records. Clinical manifestations were confirmed as follows. Pneumonia and sinusitis required radiological verification or autopsy. Meningitis was defined either by the detection of pneumococci in CSF by culture, PCR, or both methods, or by a positive blood culture in combination with clinical symptoms and an increased white blood cell count in CSF. Septic arthritis was defined by the detection of pneumococci in synovial fluid by culture, PCR, or both, whereas osteomyelitis, cellulitis, otitis media, and bronchitis were diagnosed by clinical signs and symptoms in combination with the detection of pneumococci in blood or CSF. Patients with growth of pneumococci in blood cultures but presenting without focal manifestations were classified as having bacteremia without a defined focus. Mixed episodes were classified as meningitis if meningitis coexisted with other clinical manifestations. Pneumonia was classified as such if it coexisted with other non-meningeal manifestations.

Disease severity was evaluated by the length of hospital stay and by the admission to and length of stay at an intensive care unit (ICU), respectively. IPD episodes occurring 7 days or later after hospital admission due to any other reason were defined as hospital-acquired infections.

Case fatality rate (CFR) was calculated as the proportion of patients who died within 30 days from the first sample taken in which pneumococci were detected. The mortality rate was calculated as the number of deaths in IPD per 100,000 residents per year.

### Population and prevalence data

Population data regarding age, sex, and municipality per year were collected from Statistics Sweden (SCB). Prevalence data regarding underlying diseases and conditions in the population are summarized in S1 Table. The number of individuals at risk for IPD was estimated in defined patient groups with known risk factors for IPD based on previous studies [11–19]. For chronic obstructive pulmonary disease (COPD), the estimate was based on the percentage with a Global Initiative for Obstructive Lung Disease 2019 (GOLD) score ≥2 [11].

The epidemiology of IPD has been studied in Gothenburg along with five adjacent municipalities since 1964 and in Region Västra Götaland since 1996 [20–22]. Gothenburg is the second-largest city in Sweden with 570,000 inhabitants and the provincial capital of Region Västra Götaland. The results from the present study period 2009–2019 in Region Västra Götaland were compared to data from the same region 1996–2008 [22] and to data from the Gothenburg area for the periods 1964–1980, 1981–1995, and 1996–2008 respectively [20–22]. All studies followed the same protocol, except that the current study also incorporated PCR-based diagnostics alongside culture-confirmed cases.

### Statistical analyses

Data categories are presented with numbers and percentages, and age by median, mean, and range. The Mann–Whitney U test was used to compare numerical data, Fisher’s exact test to compare proportions. Incidence rate and incidence rate ratio (IRR) for different age groups were calculated. IRR in 1996–2008 was further compared to IRR in 2009–2019 assuming Poisson distribution. The risk of IPD among people living with a specific predisposing factor was compared to the risk among all persons in the region without this factor. The risk of death within 30 days of IPD diagnosis among patients with a certain manifestation or predisposing factor was compared to the risk of death among patients without this manifestation or predisposing factor. The odds ratio of mortality for different covariates was analyzed in univariable analysis followed by multivariable logistic regression corrected for pre- and post-vaccine periods (1996–2008 and 2009–2019), predisposing factors for IPD, age, sex, and admittance to ICU.

Statistical analyses were two-sided, with alpha set at 0.05. During the analysis stage, an anonymized version of the dataset was used. Descriptive statistics were calculated using Excel 2013 (Microsoft, Redmond, WA). Multivariable analyses were performed using R Statistical Software (v4.2.2; R Core Team 2022), all other statistical analyses using GraphPad Prism version 10.2 (GraphPad Software, San Diego, CA).

## Results

### Study population and pneumococcal vaccine status

A total of 2,296 IPD episodes occurred in individuals resident in the region between 2009 and 2019. Medical records were available in 2,288 of these episodes, occurring in 2,232 individuals.

Pneumococci were isolated from blood in 2,182 episodes (95.4%), from CSF alone in 19 episodes (0.8%), and from another sterile site alone in 45 episodes (2.0%). In 42 episodes (1.8%) pneumococci were detected solely by PCR, most often from pleural fluid in patients with pneumonia complicated by empyema (*n* = 20), followed by CSF in patients with meningitis (*n* = 18).

Demographics and clinical characteristics of the patients are shown in Table 1. The median age of the patients was 69 years, with an almost equal distribution between men and women (Table 1). Fifty-five patients had more than one episode of IPD during the study period.

**Table 1:** Patient demographics and clinical characteristics in 2,288 episodes of invasive pneumococcal disease occurring in 2009–2019.

Characteristic	Episodes, n (%)
*Sex*	
Men	1,172 (51.2)
Women	1,116 (48.8)
Age in years, median (IQR) [mean; range]	69 (58–80) [65; 0–100]
*Age groups*	
0–1 year	46 (2.0)
2–17 years	58 (2.5)
18–50 years	309 (13.5)
51–64 years	472 (20.6)
65–74 years	569 (24.9)
‍75–84 years	463 (20.2)
‍‍ ≥ 85 years	371 (16.2)
*Source of infection*	
Community-acquired	2,199 (96.1)
Hospital-acquired^1^	89 (3.9)
*Number of IPD episodes per patient* ^*2*^	
One episode	2,177
Two episodes	54
Three episodes	1
*Disease severity and outcome*	
Length of hospital stay^3^ in days, median (mean) [range]	7 (12) [1–227]
ICU care	522 (22.8)
Length of stay at ICU in days, median (mean) [range]	3 (7) [0–94]
Complications	604 (26.4)
Sequelae	117 (5.1)
30-day mortality	259 (12.9)

*ICU, Intensive care unit; IPD, invasive pneumococcal disease; IQR, interquartile range.*

^1^Defined as IPD episodes occurring 7 days or later after hospital admission due to any other reason.

^2^Number refers to patients, not episodes.

^3^Length of stay in 2,252 IPD episodes; 36 additional episodes occurred in patients treated as outpatients.

Pneumococcal vaccination status was known in 29.7% (680/2,288) of all IPD episodes. Of these, previous vaccination had occurred in 129 episodes, most frequently with PPSV23 followed by PCV13 (S2 Table). A total of 88 IPD episodes occurred in 87 children <10 years of age. Among these, vaccination status was known for 55 episodes, of which 32 occurred in children who had previously received pneumococcal vaccination (S2 Table).

### Overall IPD incidence during 2009–2019

**Fig 1:**
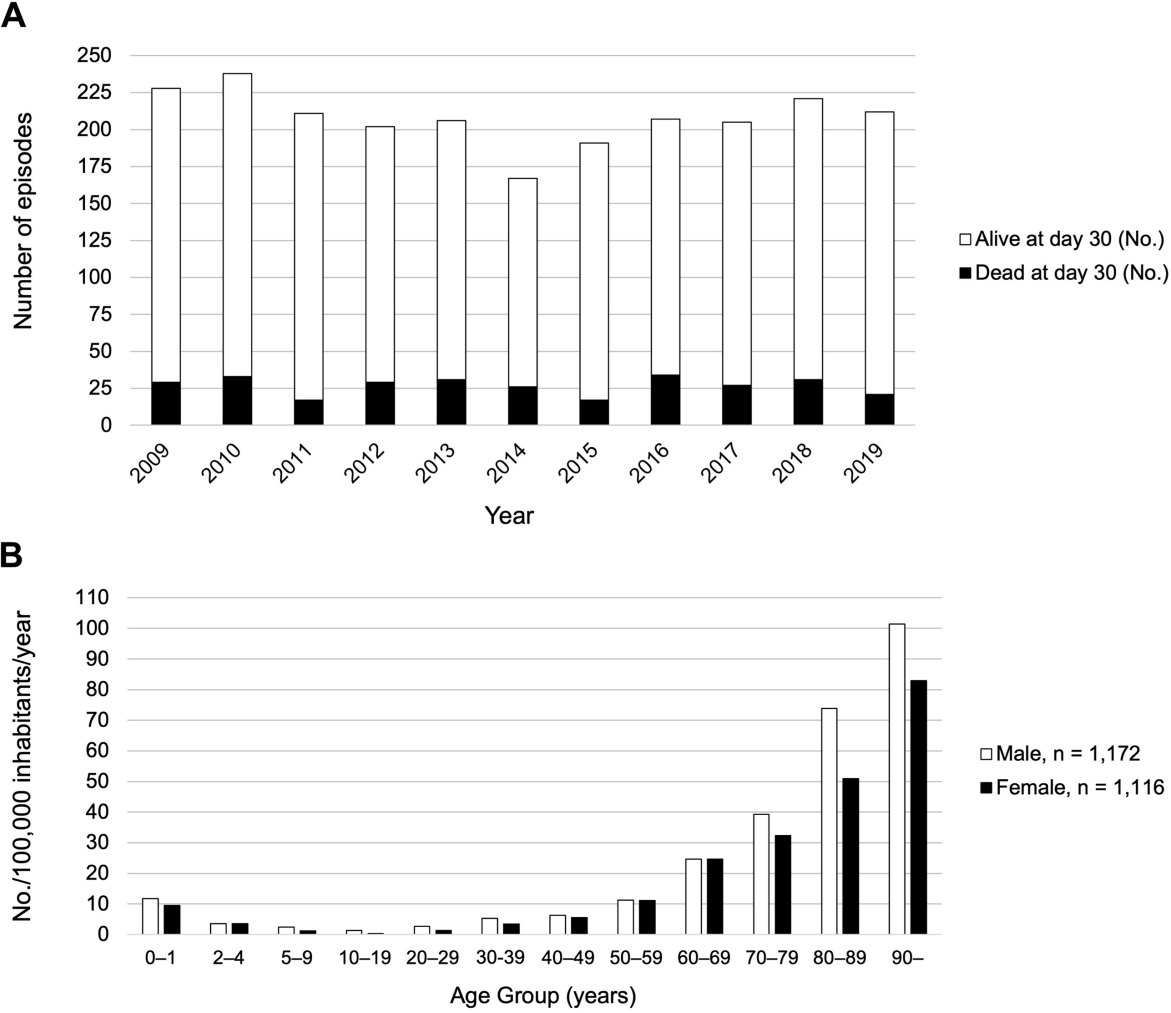
Episodes, number of deaths, and age-specific incidence rate of invasive pneumococcal disease in 2009–2019. Panel A shows the number of episodes of and deaths from invasive pneumococcal disease per year. Panel B shows the age-specific incidence rates of invasive pneumococcal disease in men and women in different age groups during 2009–2019.

The mean IPD incidence during 2009–2019 was 12.7/100,000 inhabitants/year, with a trend towards a lower incidence in the first years after PCV introduction, only to rebound in the later period (Fig 1A). The incidence was highest in the population aged ≥65 years (41.2/100,000/year). When comparing gender related IPD incidence, there was a higher incidence in males among the oldest age groups (≥70 years of age) as well as in the age groups up to 40 years (Fig 1B). In contrast, no significant difference in incidence between men and women aged 40–69 years old (Fig 1B). When analyzed across all age groups combined, there was no statistically significant difference in overall incidence between men and women (13.0 vs. 12.4/100,000; *p* = 0.27). The incidence among children 0–23 months of age was 10.7/100,000/year (Fig 1B).

### Comorbidity

In 1,765 of the IPD episodes (77%), the patient had one or more predisposing factors, most commonly cardiovascular disease (30%), chronic pulmonary disease (21%), or malignancy (20%) (Table 2). The proportion of patients with predisposing factors increased with age, from 26% (12/46) among patients 0–1 years old, 55% (32/58) among 2–17 years old, 59% (182/309) among 18–50 years old, 75% (354/472) among 51–64 years old to 84% (1,185/1,403) among patients ≥65 years old. Patients with multiple myeloma and chronic lymphocytic leukemia (CLL) had the highest incidence rates (1,497/100,000 and 505/100,000, respectively). High incidence rates were also found for patients with lung cancer and asplenia (Table 2).

**Table 2:** Predisposing factors in patients with invasive pneumococcal disease: proportion, incidence rates, and CFR.

Predisposing factor	No. of IPD episodes (%)	Incidence^1^ (No./100,000/year)	Odds ratio to get IPD (95% CI)	Died^2^ (n)	CFR^2^ (%)	Odds ratio of death (95% CI)
Cardiovascular disease	690 (30)			142	20.6	**2.45 (1.90**–3.14) ***
Pulmonary disease	472 (21)			72	15.3	1.29 (0.97–1.71)
COPD	312 (14)	71	6.32 (5.61–7.13) ***	49	15.7	1.31 (0.94–1.83)
Asthma	110 (5)	7	0.49 (0.41–0.60) ***	11	10.0	0.74 (0.41–1.38)
Malignancy, any	462 (20)	126	12.36 (11.16–13.70) ***	80	17.3	1.57 (1.19–2.08) **
Hematological	232 (10)	289	26.03 (22.68–29.87) ***	32	13.8	1.09 (0.74–1.62)
Multiple myeloma	107 (5)	1,497	147.9 (119.7–182.7) ***	17	15.9	1.29 (0.76–2.21)
Chronic lymphocytic leukemia	50 (2)	505	43.01 (32.24–57.38) ***	5	10.0	0.75 (0.32–1.81)
Solid tumors	246 (11)	86	7.54 (6.60–8.61) ***	51	20.7	1.93 (1.38–2.68) ***
Lung	57 (2)	432	36.57 (27.94–47,87) ***	16	28.1	2.73 (1.53–4.86) **
Breast	26 (1)	43	3.43 (2.33–5.05) ***	5	19.2	1.62 (0.66–4.30)
Colon	17 (1)	64	5.14 (3.18–8.29) ***	3	17.6	1.45 (0.44–4.78)
Prostate	56 (2)	66	5.35 (4.10–6.98) ***	10	17.9	1.48 (0.71–2.91)
Diabetes mellitus	359 (16)	36	3.21 (2.87–3.60) ***	49	13.6	1.08 (0.78–1.50)
Autoimmune disease	194 (8)			27	13.9	1.10 (0.71–1.67)
Rheumatoid arthritis	42 (2)	41	3.29 (2.42–4.47) ***	6	14.3	1.13 (0.51–2.59)
Polymyalgia rheumatica	14 (1)			4	28.6	2.73 (0.93–7.97)
Systemic lupus erythematosus	18 (1)	154	12.45 (7.80–19.88) ***	2	11.1	0.84 (0.19–3.38)
Liver disease	107 (5)			21	19.6	**1.70 (1.05**–2.74) *
Renal disease	127 (6)			33	26.0	**2.55 (1.66**–3.87) ***
Hemodialysis	11 (0)	202	16.30 (8.96–29.68) ***	2	18.2	1.51 (0.32–5.96)
Peritoneal dialysis	3 (0)	163	13.11 (4.18–41.09) ***	0	0.0	N.A.
Immune deficiency	107 (5)			12	11.2	0.85 (0.45–1.53)
HIV	5 (0)	54	4.30 (1.78–10.37) **	0	0.0	N.A.
Bone marrow transplant	36 (2)			3	8.3	0.61 (0.19–1.78)
Hypogammaglobulinemia	15 (1)			0	0.0	N.A.
MGUS	40 (2)	10	0.78 (0.57–1.07)	8	20.0	1.71 (0.81–3.74)
Immunosuppressive treatment^3^	294 (13)			42	14.3	1.15 (0.80–1.63)
Asplenia	45 (2)	249	20.57 (15.25–27.75) ***	9	20.0	1.71 (0.82–3.49)
Alcohol dependency	164 (7)			32	19.5	1.72 (1.14–2.59) *
Smoking^4^	412 (18)			45	10.9	0.80 (0.56–1.25)
≥1 predisposing factor	1,765 (77)			266	15.1	**3.02 (2.04**–4.55) ***
No known risk factor	523 (23)			29	5.5	
All episodes	2,288 (100)	12.7		295	12.9	

* p < 0.05, ** p < 0.01, *** *p* < 0.001

*CFR, case fatality rate; COPD, chronic obstructive pulmonary disease; HIV, human immunodeficiency virus; IPD, invasive pneumococcal disease; MGUS, monoclonal gammopathy of undetermined significance; N.A., not applicable.*

^1^Estimated annual incidence among patients living with this risk factor. For solid tumor malignancies, the estimate is based on 5-year prevalence data.

^2^Death within 30 days from sampling with the first detection of pneumococci from a sterile site.

^3^Immunosuppressive treatment includes current chemotherapy, immunosuppressants such as tumor necrosis factor inhibitors, and cortisone >2 weeks in a daily dosage equivalent to prednisolone 7.5 mg or higher.

^4^Defined as current smoking.

Among the 55 patients with two or more IPD episodes, 54 (98%) had an identifiable predisposing condition. Three of these patients had asplenia, while 25 had a hematological malignancy, primarily multiple myeloma (*n* = 15).

### Clinical manifestations

Pneumonia was the most frequent IPD manifestation, with 1,658 episodes (72%), followed by bacteremia without focus (*n* = 281, 12%) and meningitis (*n* = 179, 7.8%) (Table 3). The annual incidence of IPD with pneumonia during the study period was 9.2/100,000. The annual meningitis incidence was 1.0/100,000 in all age groups and 2.8/100,000 among children <2 years. The distribution of manifestations varied largely between the age groups. Meningitis occurred in 26% of the IPD episodes in children <2 years compared to 4.3% of the episodes in the highest age group ≥65 years (12/46 vs. 60/1,403; *p* < 0.001). Almost the opposite was found for pneumonia, which accounted for 22% of the IPD episodes in children <2 years compared to 77% among the elderly (10/46 vs. 1,085/1,403; *p* < 0.001). More rarely observed IPD manifestations, including septic arthritis (*n* = 80), acute otitis media with or without mastoiditis (*n* = 74), sinusitis (*n* = 39), endocarditis (*n* = 18), and osteitis (*n* = 17), are listed in S3 Table.

**Table 3:** Manifestations in invasive pneumococcal disease: proportions, age distribution and relative risk of death.

	No. (%) of episodes in different age groups						Fatalities		
Manifestation^1^	0–1 yr.	2–17 yrs.	18–50 yrs.	51–64 yrs.	65–100 yrs.	All ages	Died^2^ (n)	CFR^2^ (%)	Odds ratio of death (95% CI)
Pneumonia without meningitis	10 (22)	22 (38)	230 (74)	316 (67)	1,080 (77)	1,658 (72)	196	11.8%	**0.72 (0.56**–0.93) *
…with empyema	2	4	14	17	59	96	6	6.3%	
…with parapneumonic effusion	0	2	21	23	84	130	12	9.2%	
Bacteremia with no detectable focus	15 (33)	17 (29)	21 (7)	57 (12)	171 (12)	281 (12)	70	24.9%	2.63 (1.94–3.55) ***
Meningitis	12 (26)	9 (16)	39 (13)	59 (13)	60 (4)	179 (8)	17	9.5%	0.69 (0.42–1.16)
…without pneumonia	12	9	37	55	55	168	17		
…with concurrent pneumonia	0	0	2	4	5	11	0		
…with concurrent acute otitis media	1	2	14	16	15	48	4		
Other manifestations without pneumonia or meningitis	9 (20)	10 (17)	19 (6)	40 (8)	92 (7)	170 (7)	12	7.1%	**0.49 (0.27**–0.89) *
All episodes	46 (100)	58 (100)	309 (100)	472 (100)	1,403 (100)	2,288 (100)	295	12.9%	

* p < 0.05*,* *** *p* < 0.001

*CFR, case fatality rate.*

^1^In 130 episodes more than one manifestation was identified.

^2^Death within 30 days from the first sample taken in which pneumococci were identified.

A large proportion of patients diagnosed with IPD bacteremia without detectable focus had an underlying malignant disease (42.0%, 118/281), as compared to patients with bacteremic pneumonia, in whom only 18.5% (306/1,658) had known malignancy.

Hospital-acquired IPD occurred in 3.9% of all episodes (Table 1). One-third of these patients had an underlying malignant disease (34%, 30/89).

### Disease severity and complications

The patients were treated in hospital in 2,252 of the 2,288 (98.4%) IPD episodes, with a median duration of seven days (range 1–227 days) (Table 1).

In 22.8% of all episodes, the patient was admitted to the ICU (Table 1). Pneumonia was the most frequent manifestation among the ICU-admitted patients (326/522, 62.5%), followed by meningitis (*n* = 119, 22.8%) and bacteremia without focus (*n* = 46, 8.8%). Out of all patients with pneumonia, 19.7% (326/1,658) were admitted to the ICU, compared to 66.5% (119/179) of all patients with meningitis (*p* < 0.001).

In one-quarter of the IPD episodes one or more specific complications were observed, most commonly parapneumonic effusion (*n* = 135) and empyema (*n* = 98), both of which almost exclusively occurred in patients with bacteremic pneumonia (Table 1). Myocardial infarction was diagnosed in 24 IPD patients (Table 1).

One hundred and sixty-two patients out of 179 with meningitis survived (90.5%). Among these, 57 patients (35.2%), of all ages, had neurological sequelae, including paresis, hearing loss, fatigue, and loss of concentration. Among children <5 years of age with meningitis 12/14 survived, one with a subsequent facial palsy, the others without major sequelae.

### Case fatality rate

Two hundred and ninety-five patients died within 30 days of IPD diagnosis, resulting in an overall CFR of 12.9% (Table 1).

Of a total of 104 IPD episodes in children and youth <18 years, four children died (two below 12 months of age and two 3–4 years), resulting in a CFR of 3.8%. One of the children <12 months of age was known to be unvaccinated, while the other was not yet fully vaccinated and was diagnosed with congenital asplenia. Vaccination status was not known for the two older children; one of these had a severe neurological condition, and the other had no known medical conditions predisposing to IPD. CFR in different age groups is shown in S2 Fig.

CFR was higher in patients with a known predisposing risk factor or comorbidity compared to those without (15.7% vs. 5.5%; *p* < 0.001) (Table 2). Bacteremia with no detectable focus was associated with the highest CFR (24.9%), followed by pneumonia (11.8%) and meningitis (9.5%) (Table 3).

The highest CFR was seen in patients with hospital-acquired IPD (36.0%). Also, IPD with demand for ICU care was associated with a high CFR (21.1%) as well as IPD with complications (16.9%).

### Long-term trends: Comparison with previous study periods

**Fig 2:**
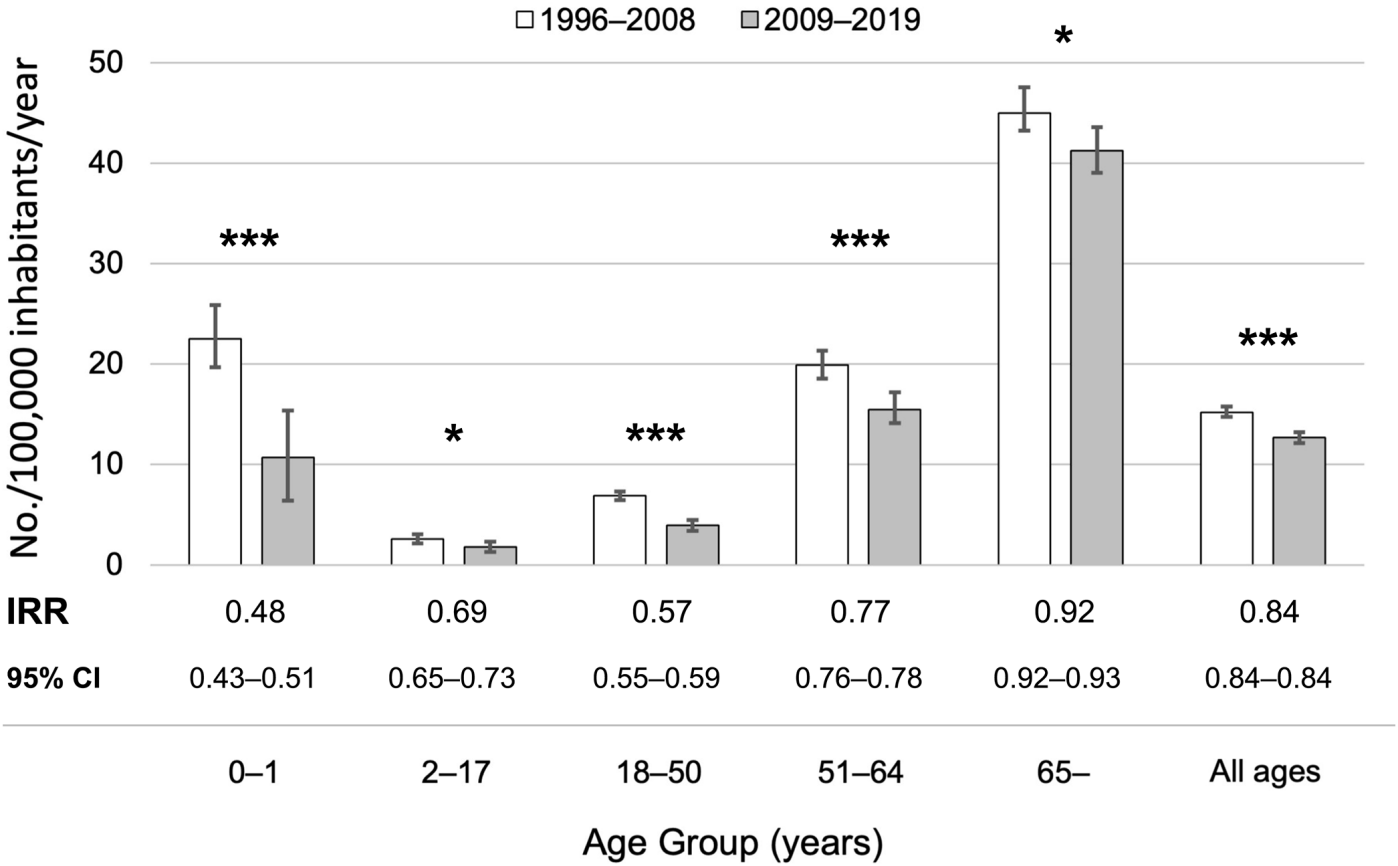
Incidence of invasive pneumococcal disease in different age groups. Study period 1996–2008 as compared to 2009–2019 in Region Västra Götaland, Sweden, presented with 95% confidence interval. * *p* < 0.05; *** *p* < 0,001. IRR, incidence rate ratio.

The IPD incidence in Region Västra Götaland was lower during the present study period (2009–2019) compared to the previous period 1996–2008 (12.7 vs. 15.1/100,000; *p* < 0.001) [22]. The incidence of IPD in the different age groups in 1996–2008 compared to 2009–2019 is shown in Fig 2.

The IPD incidence among children <2 years decreased by half during the present study period, *i.e.,* the first eleven years after the introduction of pneumococcal vaccine in the Swedish childhood vaccination program, compared to the previous period 1996–2008 (10.7/100,000 vs. 22.5/100,000; *p* < 0.001). Among the elderly ≥65 years, the incidence was 41.2/100,000 in 2009–2019 as compared to 45.0/100,000 in 1996–2008 (*p* = 0.03).

The median age at IPD diagnosis increased from 65 years in 1996–2008 to 69 years in the present study period (*p* < 0.001). During the same periods, the median age at the time of IPD diagnosis among children <2 years decreased from 414 to 344 days after birth.

Following the introduction of pneumococcal childhood vaccination, cases of pneumococcal pneumonia declined by 13%, with a mean of 174 episodes per year in 1996–2008 vs. 151 episodes per year in 2009–2019 (*p* < 0.01; S3 Fig). Although meningitis cases decreased among children aged 0–17 years, no corresponding reduction was observed in the total number of meningitis episodes across all age groups (mean 16.0 cases per year in 1996–2008 vs. 16.3 cases per year in 2009–2019).

The prevalence of underlying comorbidities or other risk factors for IPD was higher after PCV introduction compared with the previous study period (72% (1,654/2,288) vs. 67% (1,994/2,977); *p* < 0.001). Also, crude CFR was higher during the present study period (12.9% vs. 9.9%; *p* < 0.001). However, after adjusting for age, sex, underlying comorbidity, and ICU care, the difference in CFR between these two study periods was no longer statistically significant (Table 4). Length of hospital stay did not differ between the study periods (median 7 days (range 1–525) in 1996–2008 vs. 7 days (range 1–227) in 2009–2019, respectively).

**Table 4:** Case fatality rate in invasive pneumococcal disease for Region Västra Götaland in 1996–2019: Univariable model and multivariable logistic regression model.

Covariate	Crude/Unadjusted		Adjusted	
	OR (95% CI)	*P* value	OR (95% CI)	*P* value
Study period				
Pre PCV-vaccine period (1996–2008)	1.0 (Ref)		1.0 (Ref)	
Post PCV-vaccine period (2009–2019)	1.4 (1.1–1.6)	**<0.001**	1.1 (0.9–1.3)	0.32
Age group, yrs.				
0–1	1.0 (Ref)		1.0 (Ref)	
2–17	0.9 (0.2–3.7)	0.89	0.8 (0.2–3.5)	0.81
18–50	1.1 (0.4–3.1)	0.90	0.8 (0.3–2.2)	0.61
51–64	2.2 (0.8–6.1)	0.13	1.3 (0.5–3.7)	0.60
65–	6.9 (2.6–18.8)	**<0.001**	4.4 (1.6–12.1)	**0.004**
Sex				
Female	1.0 (Ref)		1.0 (Ref)	
Male	1.3 (1.1–1.5)	**0.007**	1.3 (1.0–1.5)	0.14
Predisposing factors^1^, No				
0	1.0 (Ref)		1.0 (Ref)	
1	3.0 (2.3–3.9)	**<0.001**	2.3 (1.7–3.0)	**<0.001**
2	3.3 (2.5–4.4)	**<0.001**	2.2 (1.7–3.0)	**<0.001**
3+	4.9 (3.6–6.6)	**<0.001**	3.0 (2.2–4.2)	**<0.001**
ICU care				
Not admitted	1.0 (Ref)		1.0 (Ref)	
Admitted	2.4 (2.0–2.9)	**<0.001**	2.9 (2.4–3.6)	**<0.001**
Unknown^2^	1.0 (0.8–1.4)	0.89	1.4 (1.0–1.9)	0.07

The multivariate model included all variables in the table.

*ICU, intensive care unit; OR, odds ratio; PCV, pneumococcal conjugate vaccine.*

^1^Smoking was excluded from predisposing factors due to poor data quality in the study period 1996–2008.

^2^Data missing in six episodes.

IPD data regarding all four study periods are presented in Table 5. The first two study periods included a smaller geographic area (Gothenburg with surrounding urban area) than the two following (Region Västra Götaland). To enable analysis across the entire study period, Table 5 includes data exclusively from the Gothenburg urban area. The IPD incidence was the lowest during the first study period 1964–1980 (5/100,000). CFR showed a decreasing trend during the first three study periods (from 20% in the period 1964–1980 to 9% in the period 1996–2008), again rising in the present study period as mentioned above.

**Table 5:** Incidence, CFR, and mortality of IPD in Gothenburg urban area from 1964 to 2019.

Age, years	Mean population^1^ (%)	Cases	Incidence^2^	Died^3^ (n)	CFR^3^ (%)	Mortality^4^
**1964–1980 (17 years)**						
0–1	15,248 (2.6)	52	23	8	15	3.1
2–17	118,570 (20.6)	32	2	3	9	0.1
18–50	264,282 (45.9)	161	4	28	17	0.6
51-65^a^	98,657 (17.1)	129	9	27	21	1.6
66–^a^	79,387 (13.8)	134	11	34	25	2.5
All ages	576,143 (100)	508	5	100	20	1.0
**1981–1995 (15 years)**						
0–1	15,169 (2.6)	67	29	5	7	2.2
2–17	103,624 (17.8)	35	2	3	9	0.2
18–50	280,287 (48.1)	189	4	18	10	0.4
51–64	84,475 (14.5)	167	13	27	16	2.1
65–	99,233 (17.0)	441	30	83	19	5.6
All ages	582,789 (100)	899	10	136	15	1.6
**1996–2008 (13 years)**						
0–1	11,636 (1.8)	37	24	1	3	0.66
2–17	118,604 (18.4)	31	2	1	3	0.06
18–50	308,938 (48.0)	257	6	9	4	0.22
51–64	102,280 (15.9)	228	17	10	4	0.75
65–	102,161 (15.9)	530	40	72	14	5.4
All ages	643,620 (100)	1,083	13	93	9	1.1
**2009–2019 (11 years)**						
0–1	19,212 (2.6)	21	10	1	5	0.47
2–17	130,413 (17.8)	27	2	1	4	0.07
18–50	353,285 (48.1)	128	3	4	3	0.10
51–64	115,809 (15.8)	170	13	19	11	1.5
65–	115,287 (15.7)	484	38	84	17	6.6
All ages	734,007 (100)	830	10	109	13	1.4

*CFR, case fatality rate; IPD, invasive pneumococcal disease.*

^1^Mean population during the period. Values for 1964–1980 are calculated from data obtained from 1968–1980.

^2^Number of IPD episodes/100,000 inhabitants/year.

^3^Death within 30 days from the first sample taken in which pneumococci were identified.

^4^Number of patients who died from IPD/100,000 inhabitants/year.

^a^Data from 1964–1980 includes the age groups 51–65 years and 66 years and above.

The incidence of pneumococcal meningitis in the Greater Gothenburg area has been recorded since 1970. It declined gradually from 1.4/100,000 for all ages in the period 1970–1980 to 0.9/100,000 in the present study period; for children <2 years from 12.0/100,000 to 2,8/100,000. CFR among patients with pneumococcal meningitis likewise decreased from 33% in the period 1964–1980 to 9,5% in present study period.

## Discussion

This study describes the IPD epidemiology in Southwest Sweden after the introduction of pneumococcal conjugate vaccines in the general childhood vaccination program and before the onset of the COVID-19 pandemic. It follows a series of IPD studies during 56 years in the same area [20–22].

Overall, 77% of the patients had a known medical condition predisposing to invasive pneumococcal infection. Hematological malignancies, specifically multiple myeloma and chronic lymphatic leukemia, are well-known risk factors for IPD [23]. We found a 118-fold increase in the incidence of IPD among myeloma patients compared to the overall population, and a 40-fold increase among patients with chronic lymphatic leukemia, similar to pre-PCV incidence rates [22]. Patients with multiple myeloma have impaired B- and T-cell function [24], which leaves them vulnerable to pneumococcal infections. Myeloma has been associated with repeated IPD episodes [25], which was confirmed in this study.

MGUS (monoclonal gammopathy of undetermined significance), which affects approximately 5% of the population >40 years and may develop into multiple myeloma, has been associated with an increased risk of pneumonia [26]. We did not find a greater prevalence of IPD in patients with MGUS, which might reflect an underdiagnosis of the condition in the population.

As previously described [22,23,27], high incidences of IPD were found in patients with lung cancer, asplenia, or chronic renal failure undergoing hemodialysis (34-fold, 20-fold, and 16-fold increases compared to the overall population, respectively). Only five of the 2,288 recorded IPD episodes occurred in HIV-positive patients, another well-known risk group [28]. This could be explained by the high coverage of anti-retroviral treatment in Sweden (>95% of HIV patients), resulting in immune reconstitution and a decreased susceptibility to IPD [29,30].

In almost one-fourth of all patients (23%), no predisposing factor for IPD was found. Some of these patients may have a yet unknown primary or secondary immune deficiency. Other reasons for IPD include viral coinfection, genetic predisposition, or infection by particularly virulent pneumococcal strains.

We found an equal incidence of IPD in men and women aged 40–69 years whereas men had a higher incidence than women in both the younger and older age groups. This is consistent with data from the previous study period in the same area [22], but contrasts with other international studies and earlier study periods in the region, in which men were generally overrepresented [20,21,31].

While the overall incidence in pneumonia declined, there was no substantial change in the incidence of meningitis. These conflicting results have also been observed in other parts of Europe [32]. In children <2 years, meningitis and bacteremia without focus still comprised almost two-thirds of all episodes while pneumonia was the most common IPD manifestation among the elderly, accounting for 77% of all episodes. The incidence of meningitis decreased with age, as observed in other studies [33,34].

IPD is a severe medical condition and, expectedly, we found a high rate of complications (26.4%), most commonly parapneumonic effusion and empyema in the aftermath of bacteremic pneumonia. Twenty-four patients developed a concurrent myocardial infarction, which has previously been strongly associated with IPD [35]. Similar to previous data [36] one-third of the patients with meningitis suffered from neurological sequelae, and this affected all ages.

In coherence with multiple studies from other countries [8,37,38], the overall IPD incidence among children <2 years declined substantially after PCV introduction. We also observed a general decrease in IPD in the population compared to the previous study period. The reduction of IPD incidence in the elderly was less pronounced than in infants and younger adults. This limited herd protection of the elderly after introduction of PCV in the childhood vaccination program is consistent with reports from other countries [5,39,40].

Pneumococcal serotype distribution is highly dynamic and alters over time, geographically, and after the introduction of vaccination [41,42]. Naucler *et al.* have compared IPD incidence in Swedish counties that implemented PCV13 versus PCV10 during the first seven years of the national immunization program in children (2009–2015), finding no statistically significant overall difference between those counties [43]. We and others have previously shown that IPD in persons with known predisposing factors has changed from PCV13 to non-PCV13 serotypes in Sweden [10,44]. This shift was probably a main driving force behind the lack of substantial IPD decline in the elderly and the tendency towards a rebounding incidence observed during the later part of our study period.

Despite the reduction in IPD incidence in the present study compared to the prevaccine period 1996–2008, the overall incidence of IPD has remained relatively stable in Sweden since the 1990s [9,21,22]. Besides the phenomenon of pneumococcal serotype shift this can be explained by an aging population with substantial comorbidity. Also, the number of collected blood cultures has increased significantly [45], and together with improved blood culture systems, the probability of detecting IPD episodes has increased over time. The low incidence in the first IPD study from our region (1964–1980) might be influenced by these factors.

The increase in CFR in this study compared to the previous study period in the same area was unexpected but can be attributed to the higher median age and comorbidities among the patients, as indicated in a multivariable analysis. Also, in comparison with other cohorts, the CFR in this study (12.9%) was low. In a meta-analysis of 26 studies from different countries and settings, the overall mortality was 20.8% [46]. Factors associated with death from IPD in the same study included age ≥ 65 years, nosocomial infection, underlying chronic diseases, solid organ tumors, and immunosuppressive conditions, thus similar to our findings. We did not specifically study septic shock, which was also identified as an independent risk factor for mortality, but observed a high CFR in patients admitted to the ICU (20.9%). A high prevalence of severe underlying conditions, including hematological malignancies, could explain the high CFR in patients with bacteremia without focus as well as in patients with hospital-acquired IPD (24.6% and 36.0%, respectively) in our study.

### Strengths and limitations

We here present a comprehensive study including almost all IPD episodes during an 11-year study period in a geographical area with over 1,600 000 inhabitants. The use of the same clinical protocol in previous studies allowed for long-term follow-up of IPD in the area. Data on clinical manifestations, underlying diseases, and outcomes are solid due to the high quality of the medical records. The study was terminated shortly before the COVID-19 outbreak, and our results are thus not affected by the pandemic.

Limitations of the study include lack of data regarding mechanical ventilation or septic shock management for patients receiving intensive care. Also, data on pneumococcal vaccination status were only available in one-third of all IPD episodes. Sweden has not had a national registry for systematic recording of immunization data including pneumococcal vaccination in adults, or in children outside the general childhood vaccination program, and robust data on vaccinations is thus difficult to achieve, resulting in suboptimal data quality. Furthermore, occupational exposures linked to a higher risk of IPD, such as welding, were not assessed [47]. Data on alcohol abuse and smoking were most likely underreported in the medical records; hence, the effect of these factors might be underestimated in the analysis. The case definition used in this study incorporates molecular diagnostics, which were not applied in earlier study periods. As only 1.8% of all IPD episodes were diagnosed exclusively by PCR in this study, the impact on the overall results is likely minimal. However, diagnosis by molecular analysis was more prevalent among empyema cases, which may lead to a slight overestimation of the empyema incidence in the 2009–2019 period relative to the previous period (1996–2008).

With this study, we present the follow-up data covering 56 years of continuous IPD epidemiology from a geographically relatively large area in Sweden. The present study period adds data from the first eleven years after the introduction of pneumococcal vaccination in the childhood vaccine program. With the addition of pneumococcal conjugate vaccines of higher valencies (PCV15, PCV20, and PCV21) and the implementation of a national pneumococcal vaccine program for risk groups in Sweden in 2022, further surveillance is warranted on the long-term influence of vaccination on IPD incidence and mortality, including pneumococcal serotype distribution and effects on different age groups.

## Conclusions

Following the introduction of a pneumococcal conjugate vaccine in the childhood vaccination program in 2009, IPD incidence has declined among children and adults, but to a lesser extent in the elderly >65 years of age. On the contrary, the crude CFR in IPD was higher than during the pre-vaccine period, which could be explained by increased patient age and comorbidity. The highest CFR was seen among patients with bacteremia without other clinical manifestations, in patients with hospital-acquired IPD and in IPD patients treated in the ICU. Patients with hematological malignancies, especially multiple myeloma, remain a high-risk group of IPD.

## Supporting information

S1 Fig Age structure in invasive pneumococcal disease per study period (1996–2008 vs. 2009–2019). Data shown as a violin plot where density indicates the number of episodes. Median marked as a solid vertical line, 25th and 75th quartiles marked as dotted vertical lines.
(PNG)

S2 Fig Case fatality rate (CFR) in different age groups with invasive pneumococcal disease during 2009–2019. (PNG)

S3 Fig Number of episodes and incidence of pneumonia (Panel A) and meningitis (Panel B). The dotted horizontal line shows the trend over the period. The dashed vertical line indicates the introduction of pneumococcal vaccine in the childhood vaccination program.
(PNG)

S1 Table Estimated number of individuals with different medical conditions at risk for IPD in Region Västra Götaland, Sweden, during 2009–2019. (DOCX)

S2 Table Vaccination status in different age groups. (DOCX)

S3 Table Number of IPD episodes 2009–2019 with manifestations other than pneumonia or meningitis, with age distribution. (DOCX)

## Abbreviations

CFR: Case fatality rate
CLL: Chronic lymphocytic leukemia
COPD: Chronic obstructive pulmonary disease
CSF: Cerebrospinal fluid
GOLD: Global Initiative for Obstructive Lung Disease 2019
ICU: Intensive care unit
IQR: Interquartile range
IPD: Invasive pneumococcal disease
MGUS: Monoclonal gammopathy of undetermined significance
PCR: Polymerase chain reaction
PCV: Pneumococcal conjugate vaccine
PPSV: Pneumococcal polysaccharide vaccine
SCB: Statistics Sweden
