# Supplementary figures and images for: Epidemiology of invasive pneumococcal disease in Southwest Sweden during the first eleven years after the introduction of general childhood pneumococcal vaccination
Source: PLoS One. 2026 Jun 29;21(6):e0352333. doi: 10.1371/journal.pone.0352333 (PMC13313371; doi:10.1371/journal.pone.0352333)

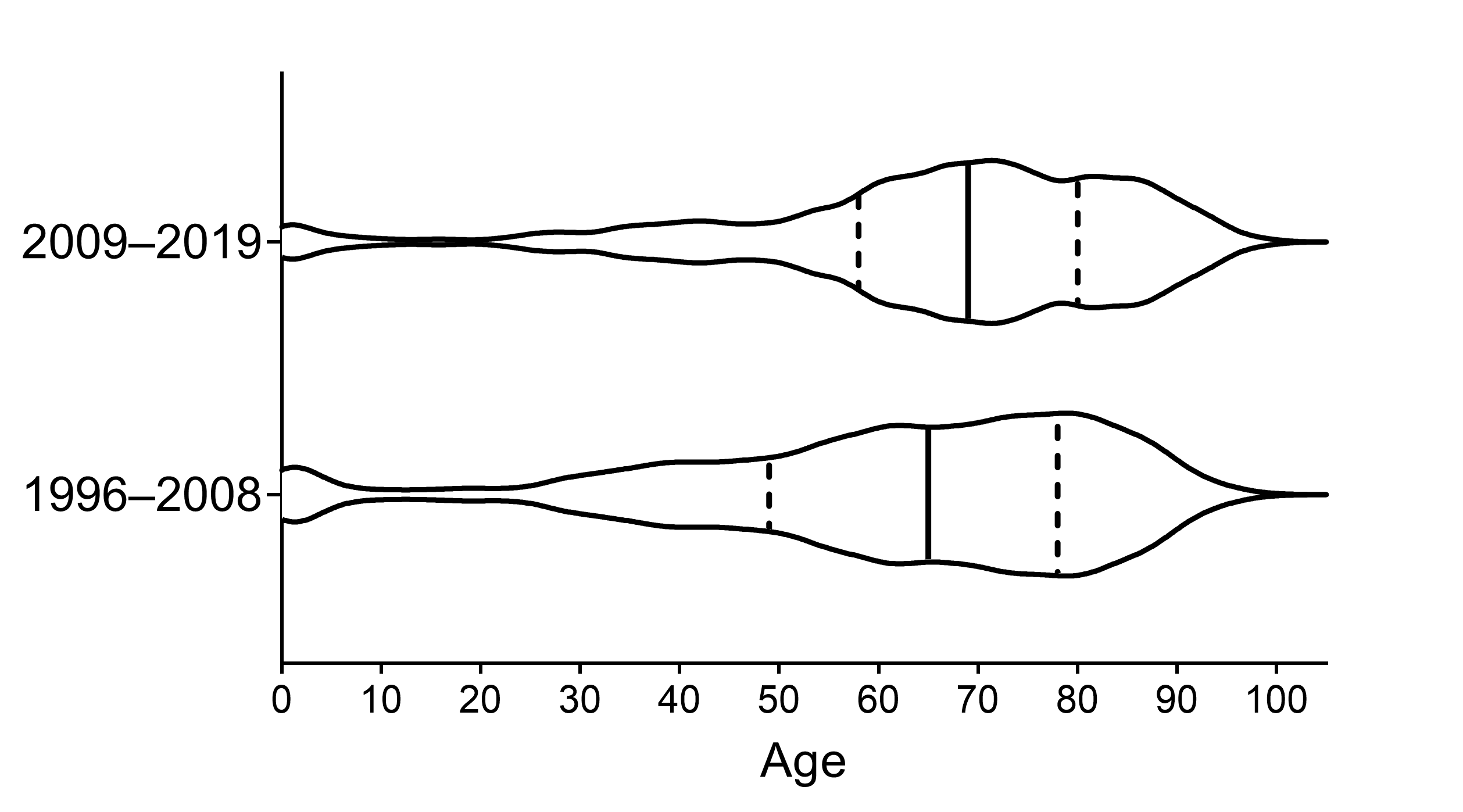

Supplement: S1 Fig — Data shown as a violin plot where density indicates the number of episodes. Median marked as a solid vertical line, 25th and 75th quartiles marked as dotted vertical lines. (PNG) [file pone.0352333.s001.png]

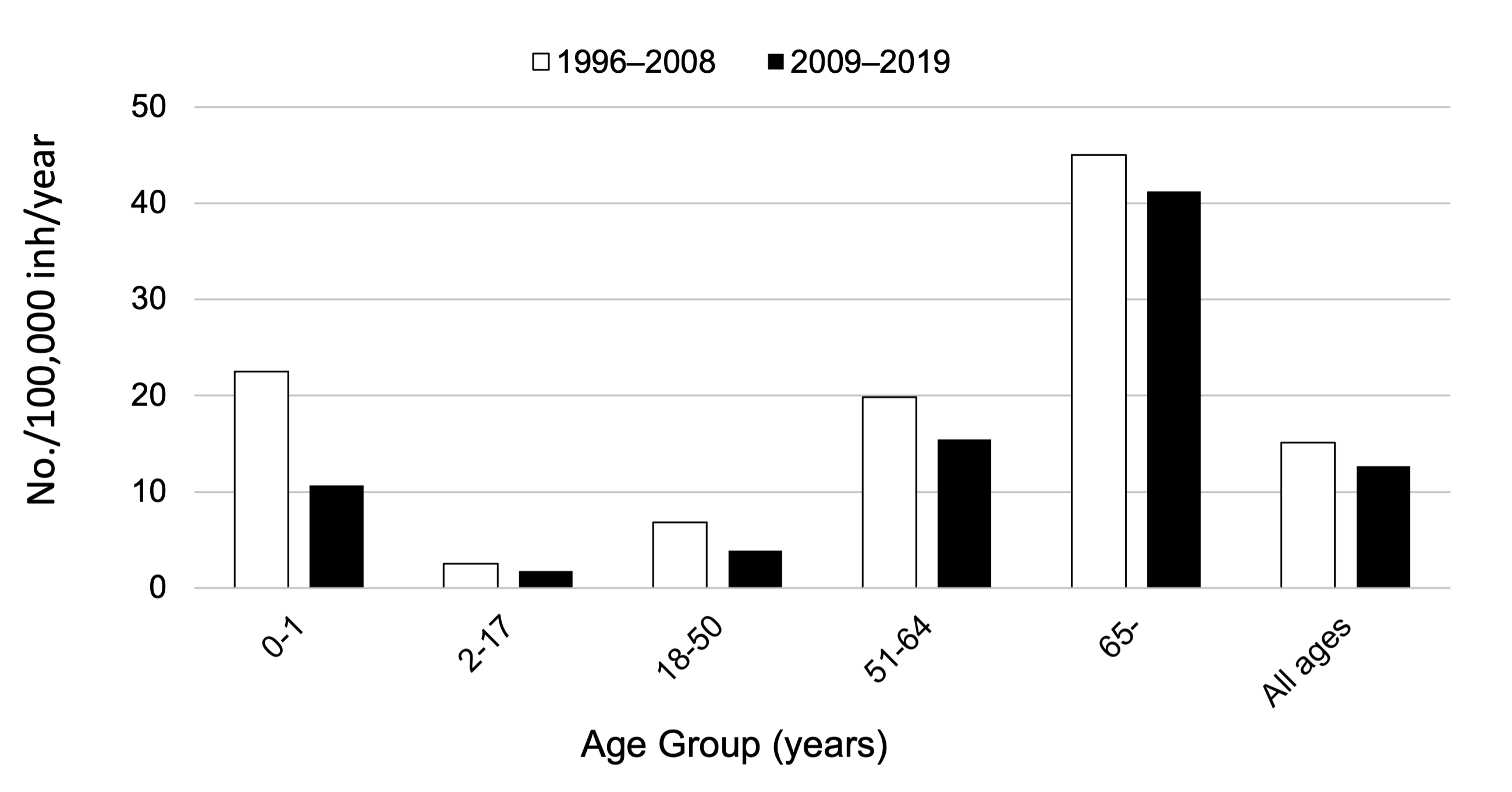

Supplement: S2 Fig — (PNG) [file pone.0352333.s002.png]

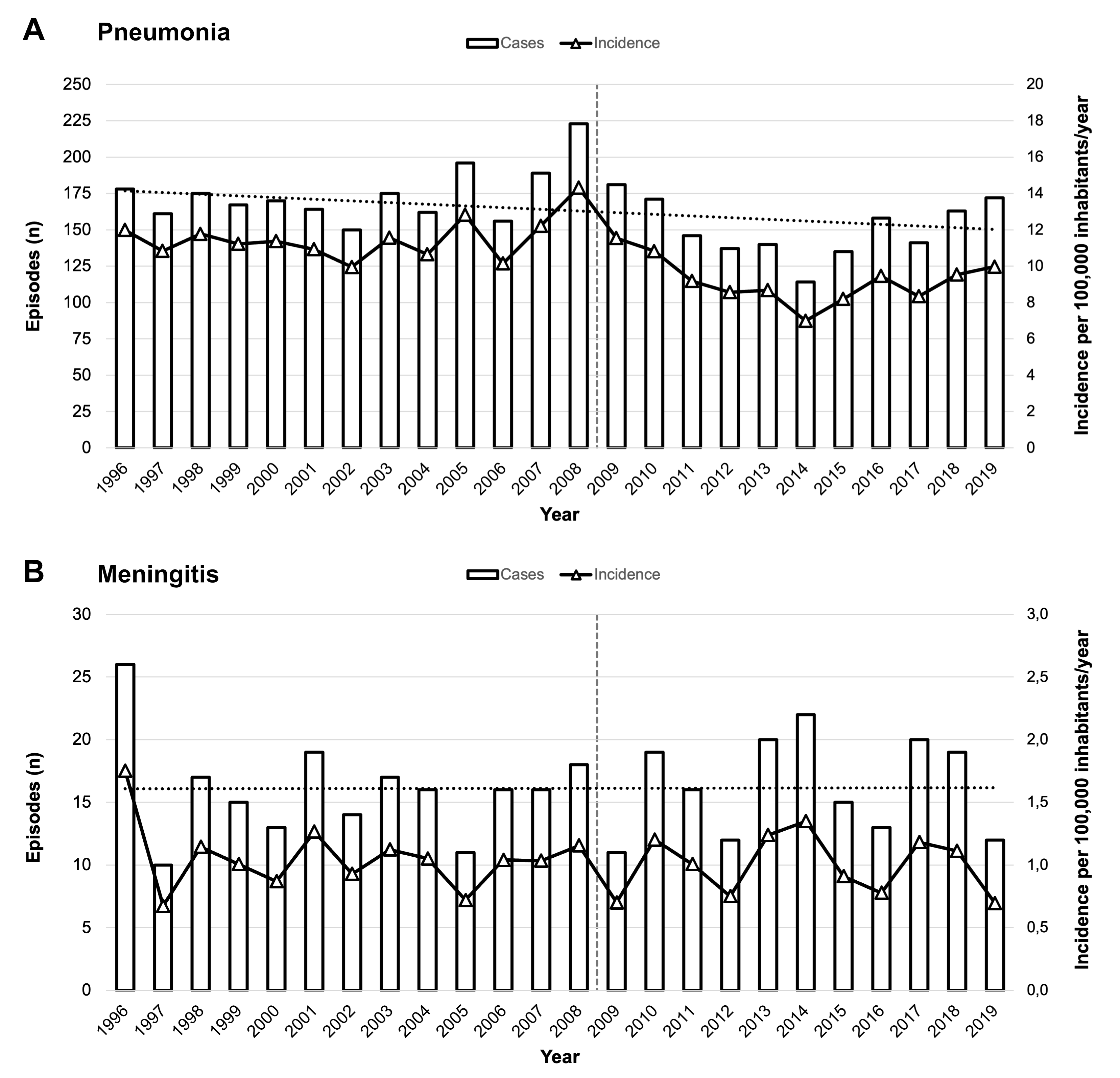

Supplement: S3 Fig — The dotted horizontal line shows the trend over the period. The dashed vertical line indicates the introduction of pneumococcal vaccine in the childhood vaccination program. (PNG) [file pone.0352333.s003.png]
