# Supplementary material for: Epidemiology of invasive pneumococcal disease in Southwest Sweden during the first eleven years after the introduction of general childhood pneumococcal vaccination
Source: PLoS One. 2026 Jun 29;21(6):e0352333. doi: 10.1371/journal.pone.0352333 (PMC13313371; doi:10.1371/journal.pone.0352333)
Supplement: S1 Table — (DOCX) [file pone.0352333.s004.docx]

**S1 Table**. **Estimated number of individuals with different medical conditions at risk for IPD in Region Västra Götaland, Sweden, during 2009–2019.**

| **Disease** | **Individuals at risk for IPD (estimated)** | **Registry, Reference** |
| --- | --- | --- |
| Chronic obstructive pulmonary disease | 40,200 | [1] |
| Asthma | 152,700 | [2-5] |
| Malignancy | 33,300 | National Cancer Register (https://www.socialstyrelsen.se/en/statistics-and-data/) |
| Hematological | 7,300 |  |
| Multiple myeloma | 650 |  |
| Chronic lymphocytic leukemia | 900 |  |
| Solid tumor^1^ | 26,000 |  |
| Lung^1^ | 1,200 |  |
| Breast^1^ | 5,500 |  |
| Colon^1^ | 2,400 |  |
| Prostate^1^ | 7,700 |  |
| Diabetes mellitus | 90,000 | Swedish National Diabetes Register (https://www.ndr.nu/) |
| Rheumatoid arthritis | 9,300 | [6] |
| Systemic lupus erythematosus | 1,060 | [7] |
| Hemodialysis^2^ | 496 | Swedish Renal Registry (https://www.medscinet.net/snr/) |
| Peritoneal dialysis^2^ | 167 |  |
| HIV^3^ | 838 | InfCareHIV Registry (https://infcarehiv.se/) |
| MGUS^4^ | 36,400 | [8] |
| Asplenia | 1,640 | [9] |

*IPD, invasive pneumococcal disease; MGUS, monoclonal gammopathy of undetermined significance.*

^1^ For solid tumors the estimate is based on 5-year prevalence data.

^2^ The average number of patients from 2009 to 2019 undergoing hemodialysis and peritoneal dialysis, respectively.

^3^ The number was calculated as the mean value of the prevalence numbers of HIV in the Region Västra Götaland for the years 2013–2019. Prevalence data for 2009–2012 were not available.

^4^ Based on a prevalence of 4.4% among adults aged 40 years and older.

1. Backman H, Vanfleteren L, Lindberg A, Ekerljung L, Stridsman C, Axelsson M, et al. Decreased COPD prevalence in Sweden after decades of decrease in smoking. Respir Res. 2020;21(1):283. Epub 20201028. doi: 10.1186/s12931-020-01536-4. PubMed PMID: 33115506; PubMed Central PMCID: PMCPMC7594463.

2. Backman H, Raisanen P, Hedman L, Stridsman C, Andersson M, Lindberg A, et al. Increased prevalence of allergic asthma from 1996 to 2006 and further to 2016-results from three population surveys. Clin Exp Allergy. 2017;47(11):1426-35. Epub 20170712. doi: 10.1111/cea.12963. PubMed PMID: 28608416.

3. Bjerg A, Sandstrom T, Lundback B, Ronmark E. Time trends in asthma and wheeze in Swedish children 1996-2006: prevalence and risk factors by sex. Allergy. 2010;65(1):48-55. Epub 20091001. doi: 10.1111/j.1398-9995.2009.02105.x. PubMed PMID: 19796226.

4. Borna E, Nwaru BI, Bjerg A, Mincheva R, Radinger M, Lundback B, et al. Changes in the prevalence of asthma and respiratory symptoms in western Sweden between 2008 and 2016. Allergy. 2019;74(9):1703-15. Epub 20190701. doi: 10.1111/all.13840. PubMed PMID: 31021427.

5. Hicke-Roberts A, Aberg N, Wennergren G, Hesselmar B. Allergic rhinoconjunctivitis continued to increase in Swedish children up to 2007, but asthma and eczema levelled off from 1991. Acta Paediatr. 2017;106(1):75-80. Epub 20160520. doi: 10.1111/apa.13433. PubMed PMID: 27102081.

6. Neovius M, Simard JF, Askling J, group As. Nationwide prevalence of rheumatoid arthritis and penetration of disease-modifying drugs in Sweden. Ann Rheum Dis. 2011;70(4):624-9. doi: 10.1136/ard.2010.133371. PubMed PMID: 21149495.

7. Ingvarsson RF, Bengtsson AA, Jonsen A. Variations in the epidemiology of systemic lupus erythematosus in southern Sweden. Lupus. 2016;25(7):772-80. doi: 10.1177/0961203316635288. PubMed PMID: 26923282.

8. Sigurbergsdottir AY, Rognvaldsson S, Thorsteinsdottir S, Sverrisdottir I, Sigurethardottir GA, Vietharsson B, et al. Disease associations with monoclonal gammopathy of undetermined significance can only be evaluated using screened cohorts: results from the population-based iStopMM study. Haematologica. 2023;108(12):3392-8. Epub 20231201. doi: 10.3324/haematol.2023.283191. PubMed PMID: 37439374; PubMed Central PMCID: PMCPMC10690899.

9. Kanhutu K, Jones P, Cheng AC, Grannell L, Best E, Spelman D. Spleen Australia guidelines for the prevention of sepsis in patients with asplenia and hyposplenism in Australia and New Zealand. Intern Med J. 2017;47(8):848-55. doi: 10.1111/imj.13348. PubMed PMID: 27925427.
