# Supplementary material for: Epidemiology of invasive pneumococcal disease in Southwest Sweden during the first eleven years after the introduction of general childhood pneumococcal vaccination
Source: PLoS One. 2026 Jun 29;21(6):e0352333. doi: 10.1371/journal.pone.0352333 (PMC13313371; doi:10.1371/journal.pone.0352333)
Supplement: S2 Table — (DOCX) [file pone.0352333.s005.docx]

**S2 Table. Vaccination status in different age groups.**

| **Vaccination status and vaccine type** | **<10 years** | **<18 years** | **‍≥65 years** | **All ages** |
| --- | --- | --- | --- | --- |
| ‍Patients, n | 87 | 102 | 1,364 | 2,232 |
| ‍IPD episodes, n | 88 | 104 | 1,403 | 2,288 |
| ‍Pneumococcal vaccination status known, n (%) episodes | 55 (62.5) | 61 (58.7) | 358 (25.5) | 682 (29.8) |
| ‍Vaccinated, n (% of known episodes) | 32 (58.2) | 32 (52.5) | 69 (19.3) | 129 (18.9) |
| ‍PPSV23 only, n | 0 | 0 | 28 | 40 |
| ‍PCV7 only, n | 5 | 5 | 0 | 5 |
| ‍PCV10 only, n | 5 | 5 | 0 | 5 |
| ‍PCV13 only, n | 12 | 12 | 3 | 18 |
| ‍PPSV23 + PCV7, n | 0 | 0 | 0 | 1 |
| ‍PPSV23 + PCV10, n | 0 | 0 | 0 | 0 |
| ‍PPSV23 + PCV13, n | 2 | 2 | 5 | 8 |
| ‍Vaccine type unknown, n | 8 | 8 | 33 | 52 |
| ‍Not vaccinated, n (% of known episodes) | 23 (41.8) | 29 (47.5) | 289 (80.7) | 553 (81.1) |

*IPD, invasive pneumococcal disease; PPSV, Pneumococcal polysaccharide vaccine; PCV, Pneumococcal conjugate vaccine.*
