# Supplementary material for: Epidemiology of invasive pneumococcal disease in Southwest Sweden during the first eleven years after the introduction of general childhood pneumococcal vaccination
Source: PLoS One. 2026 Jun 29;21(6):e0352333. doi: 10.1371/journal.pone.0352333 (PMC13313371; doi:10.1371/journal.pone.0352333)
Supplement: S3 Table — (DOCX) [file pone.0352333.s006.docx]

**S3 Table. Number of IPD episodes 2009–2019 with manifestations other than pneumonia or meningitis, with age distribution.**

| **Other manifestations^1^** | **Number of episodes in different age groups** | | | | | |
| --- | --- | --- | --- | --- | --- | --- |
|  | **0–1 yr.** | **2–17 yrs.** | **18–50 yrs.** | **51–64 yrs.** | **65–100 yrs.** | **All ages** |
| Upper respiratory tract infections (URTI) | | | | | | |
| Acute otitis media and mastoiditis | 8 | 8 | 15 | 20 | 23 | 74 |
| Sinusitis | 1 | 4 | 6 | 13 | 15 | 39 |
| Other URTI | 1 | 0 | 1 | 4 | 11 | 17 |
| Epiglottitis | 0 | 0 | 1 | 1 | 5 | 7 |
| Skin, bone, joint and soft tissue | | | | | | |
| Septic arthritis | 0 | 0 | 4 | 19 | 57 | 80 |
| Erysipelas, cellulitis, phlegmon, abscess | 2 | 1 | 3 | 10 | 7 | 23 |
| Osteitis | 0 | 0 | 0 | 9 | 8 | 17 |
| Intra-abdominal | | | | | | |
| Peritonitis | 0 | 0 | 4 | 2 | 3 | 9 |
| Cholecystitis, cholangitis | 0 | 0 | 2 | 0 | 2 | 4 |
| Mycotic aneurysm | 0 | 0 | 0 | 2 | 2 | 4 |
| Intra-abdominal abscess | 0 | 0 | 0 | 2 | 1 | 3 |
| Salpingitis, tubo-ovarian abscess | 0 | 0 | 2 | 0 | 0 | 2 |
| Appendicitis | 0 | 0 | 1 | 0 | 0 | 1 |
| Endocarditis | 0 | 0 | 2 | 9 | 7 | 18 |
| Acute bronchitis | 0 | 0 | 1 | 1 | 7 | 9 |
| Catheter-related infection | 0 | 3 | 0 | 0 | 0 | 3 |
| Endophthalmitis | 0 | 0 | 0 | 0 | 3 | 3 |
| Number of episodes | 11 | 15 | 39 | 77 | 141 | 283 |

*IPD, invasive pneumococcal disease; URTI, upper respiratory tract infection.*

^1^ In 25 episodes, more than one manifestation other than pneumonia or meningitis was detectable.
